# Vascular endothelial growth factor as a non-invasive marker of pulmonary vascular remodeling in patients with bronchitis-type of COPD

**DOI:** 10.1186/1465-9921-8-22

**Published:** 2007-03-08

**Authors:** Hiroshi Kanazawa, Kazuhisa Asai, Saeko Nomura

**Affiliations:** 1 Department of Respiratory Medicine, Graduate School of Medicine, Osaka City University, 1-4-3, Asahi-machi, Abenoku, Osaka, 545-8585, Japan

## Abstract

**Background:**

Several studies have indicated that one of the most potent mediators involved in pulmonary vascular remodeling is vascular endothelial growth factor (VEGF). This study was designed to determine whether airway VEGF level reflects pulmonary vascular remodeling in patients with bronchitis-type of COPD.

**Methods:**

VEGF levels in induced sputum were examined in 23 control subjects (12 non-smokers and 11 ex-smokers) and 29 patients with bronchitis-type of COPD. All bronchitis-type patients performed exercise testing with right heart catheterization.

**Results:**

The mean pulmonary arterial pressure (mPAP) and pulmonary vascular resistance (PVR) after exercise were markedly increased in all bronchitis-type patients. However, both parameters after exercise with breathing of oxygen was significantly lower than in those with breathing of room air. To attenuate the effect of hypoxia-induced pulmonary vasoconstriction during exercise, we used the change in mPAP or PVR during exercise with breathing of oxygen as a parameter of pulmonary vascular remodeling. Change in mPAP was significantly correlated with VEGF level in induced sputum from patients with chronic bronchitis (r = 0.73, p = 0.0001). Moreover, change in PVR was also correlated with VEGF level in those patients (r = 0.57, p = 0.003).

**Conclusion:**

A close correlation between magnitude of pulmonary hypertension with exercise and VEGF level in bronchitis-type patients could be observed. Therefore, these findings suggest the possibility that VEGF level in induced sputum is a non-invasive marker of pulmonary vascular remodeling in patients with bronchitis-type of COPD.

## Background

Pulmonary vascular remodeling leading to pulmonary hypertension is a characteristic feature of chronic obstructive pulmonary disease (COPD), and has been associated with the development of COPD [1]. In agreement with this notion, previous studies have suggested that the natural history of pulmonary hypertension in COPD might commence at moderate degrees of disease severity [2]. In fact, hypoxia has been classically considered the major pathogenic mechanism of pulmonary vascular remodeling in COPD. However, structural abnormalities of pulmonary arteries are not exclusive of advanced COPD, since they have been shown also in patients with mild COPD without arterial hypoxemia and even in smokers with normal lung function [3]. Wright et al. also found an increase in wall area of small pulmonary vessels by intimal thickening in patients with mild to moderate COPD and medial thickening in severe cases [4]. Thus, since patients with mild COPD are not usually hypoxemic, the etiology of pulmonary vascular remodeling remains uncertain.

One important pathological feature of COPD is chronic inflammation characterized by an influx of inflammatory cells, predominantly neutrophils, macrophages, and CD8+ T lymphocytes, into the airway walls and parenchyma [5]. COPD, a syndrome of variable phenotype, is mostly caused by inhaled cigarette smoke. Over time, alveolar destruction results in emphysema, and chronic airway inflammation leads to chronic bronchitis. Chronic bronchitis is a clinical syndrome defined by chronic cough and sputum production. In chronic bronchitis, airway inflammation is associated with structural alterations including an increase in the amount of smooth muscle and connective tissue in the airway wall [6]. Moreover, previous observations have indicated that pulmonary arteries in patients with chronic bronchitis have increased adventitial infiltration of activated T lymphocytes [7,8]. Therefore, active airway inflammation might affect pulmonary vascular remodeling in chronic bronchitis. In contrast, it has been supposed that emphysema may lead to loss of the pulmonary vascular bed [9]. The Global Initiative for Obstructive Lung Disease (GOLD) Workshop Report defines COPD as a disease state characteristics by airflow limitation that is not fully reversible [10]. This chronic airflow limitation characteristic of COPD is attributed to a mixture of two manifestations involved in COPD: parenchymal destruction (emphysema) and small airway disease (obstructive bronchiolitis). The GOLD definition differs from many previous definitions of COPD, which emphasized the terms of emphysema and chronic bronchitis. According to GOLD, the relative contribution of parenchymal destruction and small airway disease toward airflow limitation varies in individuals.

A previous study revealed endothelial dysfunction in pulmonary arteries of patients with mild COPD [11]. Subjects with greater impairment of endothelial function had more pronounced pulmonary vascular remodeling, such as intimal thickening. Because the endothelium plays a central role in regulating vascular tone and controlling cell growth, the impairment of endothelial function might promote pulmonary vascular remodeling at this early stage of COPD. To date, it is not known which mediators are involved in this process. However, several studies have indicated that one of the most potent mediators involved in pulmonary vascular remodeling is vascular endothelial growth factor (VEGF) [12]. VEGF promotes an array of responses in endothelial cell proliferation and angiogenesis with new vessel formation *in vivo *[13]. These findings may suggest the potential roles of VEGF in the pathogenesis of pulmonary vascular remodeling. Therefore, we attempted to determine whether airway VEGF level reflects pulmonary vascular remodeling in patients with bronchitis-type of COPD.

## Methods

### Subjects

All COPD patients satisfied the GOLD criteria for the diagnosis, and were selected from the respiratory outpatient clinic of our institution. They have undergone the evaluation for low attenuation area (LAA) on high-resolution computed tomographic scans (HRCT) of the lungs prior to the entry of this study. Four slices 1 mm thick were obtained at three anatomical levels at full inspiration, that is, near the superior margin of the aortic arch (level of the upper lung field), at the level of the carina (level of the middle lung field), and at the level of the orifice of the inferior pulmonary veins (level of the lower lung field). LAA were scored visually in each bilateral lung field according to the method of Goddard et al. [14]. Total scores were calculated and the severity of emphysema was graded as follows; score 0, LAA < 5%; score 1, 5% < LAA < 25%; score 2, 25% < LAA < 50%; score 3, 50% < LAA < 75%; score 4, LAA > 75%. Grade 0, total score = 0; grade 1, total score = 1–6, grade 2, total score = 7–12; grade 3, total score = 13–18; grade 4, total score = 19–24. HRCT images were analyzed independently by two chest physicians with no knowledge of the clinical information. The patients were classified according to the visual HRCT findings as follows: absence of emphysema, which showed little emphysema and LAA grade < 1, reflecting small airway disease (bronchitis-type), and presence of emphysema, which showed apparent emphysema > grade 2, reflecting parenchymal destruction (emphysema-type). Thus, bronchitis-type of COPD was defined as cough and sputum production occurring on most days of the month for at least 3 months a year during the 2 years prior to the study and on the basis of the HRCT findings (HRCT total score < 6). Finally, 29 patients with bronchitis-type of COPD (classification of severity in GOLD: 3 mild, 23 moderate, and 3 severe) and 23 normal control subjects (12 non-smokers and 11 ex-smokers) were included in the study. All control subjects were healthy, who had no history of respiratory disease. Bronchitis-type patients had no exacerbation, which were defined as increased dyspnea associated with a change in the quality and quantity of sputum that led the subject to seek medical attention, during the 1 month preceding the study. All patients had been free of acute upper respiratory tract infections and none had received glucocorticoids or antibiotics within the 1 month preceding the study, or bronchodilators within the previous 48 hours of exercise testing. Their regular medication consisted of inhaled short-acting anticholinergic agents and the beta2-agonist salbutamol on demand.

The subjects were non-atopic (i.e., they had negative skin tests for common allergen extracts) and had no past history of asthma or allergic rhinitis. Pulmonary function tests including the diffusing capacity of the lung for carbon monoxide (Dlco) were performed within the 1 week before this study. Patients with evidence of coronary artery disease, valvular heart disease, systemic hypertension, or primary myocardial disease were excluded from the study. None of the patients had radiological or clinical evidence of pulmonary congestion or right heart failure. Concomitant left ventricular dysfunction was excluded in all patients by echocardiography and determination of pulmonary wedge pressure (PWP). No subjects in this study were included as subjects in our previous study. All patients gave their written informed consent for participation in this study, which was approved by the Ethics Committee of Osaka City University, Japan. This investigation conforms with the principles outlined in the Declaration of Helsinki.

### Exercise test

On the first day of the study, all bronchitis-type patients underwent a progressive incremental exercise test while sitting on an ergometer (EM840; Siemens, Germany), starting at 0 W for 3 minutes and adding 10 W every minute until the symptom-limited maximum was reached, as we previously described [15]. The purpose of the incremental exercise test was to determine the maximal exercise capacity. On the day following the test, all patients underwent right heart catheterization. A balloon-tipped pulmonary arterial catheter was advanced to the pulmonary artery for measurement of pulmonary arterial pressure (PAP) and PWP. In addition, a plastic catheter was placed into the brachial artery to monitor systemic arterial pressure and to sample systemic arterial blood. Cardiac output (CO) was determined by the thermodilution method, using a Fukuda Denshi CO computer. Arterial oxygen tension (PaO2) was measured with a blood gas analyzer (Model IL 1312; Instrumentation Laboratory). Resting hemodynamic and blood gas data were obtained about 20 minutes after the patient had been seated comfortably on the ergometer. Each patient then performed a constant-load exercise test for 5 minutes while on the ergometer at a workload corresponding to 60% of the previously determined maximal workload. Hemodynamic and blood gas measurements were made during the final minute of constant-load exercise. After exercise with breathing of room air, 100% oxygen was given to the patient for 60 minutes via nasal cannula at a rate of 3 L/min. All of the protocols described above were repeated while the patient breathed oxygen.

### Calculations

From parameters directly measured, the following indices were derived:

Cardiac index (CI) (L/min/m2) = Cardiac output/Body surface area,

Pulmonary vascular resistance (PVR) (mmHg/L/min/m2) = (PAP - PWP)/CI

### Sputum induction and processing

Sputum induction was performed three days after the exercise challenge test, as we previously described [16]. The sputum sample diluted with phosphate-buffered solution containing dithiothreitol (a final concentration of 1 mM) was then centrifuged at 400 g for 10 minutes. The supernatant was stored at -70°C for subsequent assay of VEGF. VEGF concentration was measured with an enzyme-linked immunosorbent assay kit (R&D system Inc, Minneapolis, MN, USA). The minimum detectable level of VEGF in this assay system is 5.0 pg/mL. All subjects produced an adequate specimen of sputum; a sample was considered adequate if the patient was able to expectorate at least 2 mL of sputum.

### Statistical analysis

All values are presented as mean (SD). Multiple comparisons were performed by one-way analysis of variance (ANOVA). When ANOVA revealed a significant difference, the Bonferroni correction was applied. The significance of correlation was evaluated by determining Spearman's rank correlation coefficients. A p value of less than 0.05 was considered significant.

## Results

The clinical characteristics of the 23 control subjects and 29 patients with bronchitis-type of COPD are summarized in Table 1. The three groups were well matched with respect to age. However, baseline FEV1 and FEV1/forced vital capacity was significantly lower in bronchitis-type patients than in control subjects, and Dlco was also decreased in bronchitis-type patients. In contrast, the HRCT score was significantly higher in bronchitis-type patients than in control subjects. VEGF levels in induced sputum were also significantly higher in patients with bronchitis-type patients than in control subjects.

**Table 1:** Clinical characteristics of study subjects

	Normal controls		Chronic bronchitis
	non-smoker	ex-smoker	
Patient number (male/female)	12 (12/0)	11 (11/0)	29 (29/0)
Age (years)	51.5 (4.5)	55.5 (3.5)	62.0 (5.7)
Smoking (pack-years)	0	26.9 (4.3)	30.7 (3.6)
FEV1 (% predicted)	89.5 (5.5)	83.4 (2.9)	63.7 (8.2)*
FEV1/FVC (%)	80.5 (5.4)	78.9 (3.0)	61.4 (4.9)*
DLCO (%)	97.5 (2.5)	90.5 (3.5)	64.0 (8.0)*
HRCT score	0	1.4 (1.1)*	4.2 (1.3)*
VEGF in sputum (pg/mL)	1950 (950)	1760 (920)	3500 (1070)*

Abbreviations: FEV1 = forced expiratory volume in 1 second; FVC = forced vital capacity;

DLCO = diffusing capacity of lung for carbon monoxide; HRCT = high-resolution computed tomographic scans;

VEGF = vascular endothelial growth factor.

* p < 0.01 compared with normal controls. Data are presented as mean (SD).

**Figure 1:**
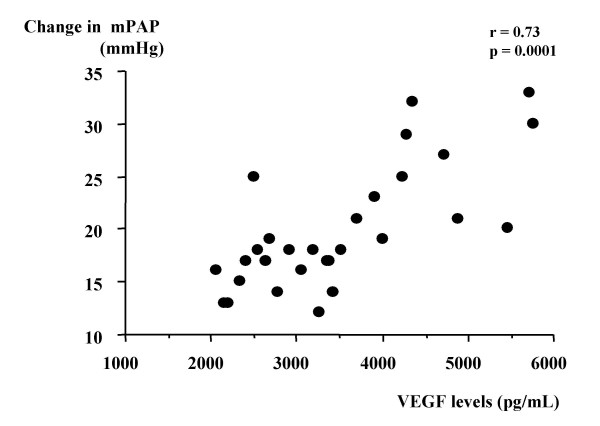
Correlation between VEGF level in induced sputum and change in mPAP during exercise with breathing of oxygen in patients with bronchitis-type of COPD.

**Figure 2:**
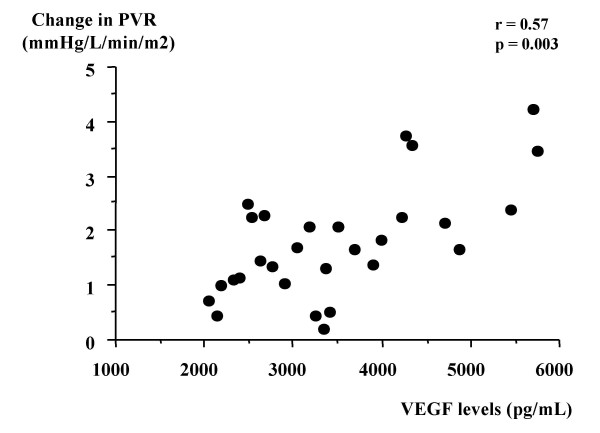
Correlation between VEGF level in induced sputum and change in PVR during exercise with breathing of oxygen in patients with bronchitis-type of COPD.

Table 2 shows hemodynamic parameters at rest and after exercise with breathing of room air or oxygen in bronchitis-type patients. Neither heart rate nor mean arterial pressure at rest or after exercise differed significantly between breathing of room air and breathing of oxygen. We determined that exercise-induced hypoxemia was attenuated by breathing of oxygen in all patients (PaO2 after exercise without oxygen: range 41–60 mmHg; PaO2 after exercise with oxygen: range 79–94 mmHg). Neither mPAP nor PVR at rest differed significantly between breathing of room air and breathing of oxygen. The mPAP and PVR after exercise were markedly increased in all patients. However, the mPAP and PVR after exercise with breathing of oxygen was significantly lower than in those with breathing of room air (mPAP: p = 0.0009, PVR: p = 0.03). To attenuate the effect of hypoxia-induced pulmonary vasoconstriction with exercise, we used the change in mPAP or PVR during exercise with breathing of oxygen as a parameter of pulmonary vascular remodeling. Change in mPAP was significantly correlated with VEGF level in induced sputum from bronchitis-type patients (r = 0.73, p = 0.0001) (Fig 1). Moreover, change in PVR was also correlated with VEGF level in those patients (r = 0.57, p = 0.003) (Fig 2).

**Table 2:** Hemodynamic parameters at rest and after exercise in patients with bronchitis-type of COPD

	Oxygenation (-)	Oxygenation (+)
HR		
rest	77 (11)	76 (11)
after exercise	116 (17)	114 (14)
MAP (mmHg)		
rest	93 (8)	92 (7)
after exercise	140 (13)	135 (11)
PaO2 (mmHg)		
rest	76 (5)	111 (6)**
after exercise	50 (6)	85 (4)**
mPAP (mmHg)		
rest	22.9 (1.9)	21.9 (2.0)
after exercise	46.0 (6.2)	41.8 (6.4)**
PVR (mmHg/L/min/m2)		
rest	5.97 (0.76)	5.91 (0.81)
after exercise	8.28 (1.21)	7.67 (1.27)*

Abbreviations: HR = heart rate; MAP = mean arterial pressure; PaO2 = arterial oxygen tension;

mPAP = mean pulmonary arterial pressure; PVR = pulmonary vascular resistance.

** p < 0.01, * p < 0.05 compared with Oxygenation (-).

Data are presented as mean (SD).

## Discussion

The novel aspect of this investigation is the finding of a close correlation between change in magnitude of pulmonary hypertension from pre- to post-exercise and VEGF level in sputum samples from bronchitis-type patients. All previous studies investigating pulmonary vascular remodeling were based on an invasive pathologic examination by using the resected lung specimens. Therefore, we attempted non-invasive analysis of the degree of pulmonary vascular remodeling. However, to the best of our knowledge, no information is available on potential parameters of pulmonary vascular remodeling derived from physiological and biochemical characterization of human subjects. In this study, pulmonary hypertension became particularly pronounced in bronchitis-type patients during exercise, indicating that the increase in pulmonary blood flow and hypoxemia during exercise resulted in an exaggerated pulmonary hypertension. For this reason, it appeared that bronchitis-type patients had the ability to accommodate to pulmonary blood flow at rest, but that they had lost the ability to accommodate increased pulmonary blood flow through vascular remodeling and hypoxic pulmonary vasconstriction with exercise [17]. To enable testing with an attenuated degree of hypoxic pulmonary vasoconstriction during exercise, we have performed exercise challenge with breathing of oxygen. In fact, we found that mPAP and PVR after exercise with breathing of oxygen were significantly lower than those with breathing of room air. Thus, we determined that exercise challenge testing with breathing of oxygen is a reliable method for evaluation of degree of pulmonary vascular remodeling in bronchitis-type patients. Using both pulmonary hemodynamic study and histologically morphological analysis, Kubo and their colleagues have also reported that pulmonary vascular remodeling is closely related to exercise-induced pulmonary hypertension [18]. However, our method is also invasive, since all subjects underwent right heart catheterization followed by an exercise test. Therefore, a non-invasive marker of pulmonary vascular remodeling will be required to be widely used in the clinical investigation in this field.

Cigarette smoking causes an inflammatory reaction in the arteries of patients with chronic bronchitis. Accordingly, we hypothesized that the vascular remodeling in pulmonary arteries with bronchitis-type of COPD could be related to an inflammatory process. Indeed, airway inflammation has been shown to be involved in the pathogenesis of some forms of pulmonary hypertension [19]. Several studies performed in lungs of patients with mild COPD have shown apparent abnormalities in the structure of the pulmonary arteries, in most cases consisting of the thickening of the intimal layer [20]. Interestingly, the intensity of the intimal thickening has been shown to correlate with the severity of the inflammatory infiltrates in small airways, suggesting that an inflammatory process might also account for the vascular remodeling of pulmonary arteries [21,22]. A potential mechanism for the increased number of inflammatory cells in pulmonary arteries of patients with bronchitis-type of COPD could be their migration from adjacent bronchioles. However, the precise mechanism by which inflammatory cells may induce pulmonary vascular remodeling remains unknown. One possibility is that inflammatory cells constitute a source of cytokines and growth factors such as VEGF, that may target the endothelial cells and contribute to the development of structural and functional abnormalities of the vessel walls [23]. In the present study we found that airway VEGF might influence pulmonary vascular remodeling. These findings suggest that increased VEGF level contributes to increased and abnormal proliferation of endothelial and vascular smooth muscle cells in pulmonary vessels, leading to vascular remodeling. Indeed, VEGF has been found to be involved in vascular remodeling in primary pulmonary hypertension, which is characterized by endothelial and smooth muscle proliferation [24]. Cigarette smoking may up-regulate the expression of VEGF, as suggested by acute increase in VEGF levels during smoking [25]. Accordingly, it has been supposed that VEGF could play a role in the pathogenesis of the endothelial cell proliferation shown in pulmonary arteries of smokers. Interestingly, it has been suggested that decrease in VEGF might be involved in the pathogenesis of emphysema through apoptotic mechanisms of pulmonary endothelial cells [26]. In contrast, high levels of VEGF induced airway remodeling in bronchitis-type of COPD [27]. These previous findings have promoted a growing interest in clarifying that VEGF may affect pulmonary vascular remodeling of these common types of COPD. With this background in mind, our findings indicate the potential role of VEGF in the pathogenesis of the vascular changes that take place in bronchitis-type of COPD.

We suggested the significant association between airway VEGF level and degree of pulmonary vascular remodeling. The high levels of VEGF receptors expression in the pulmonary vessels were observed in the vascular smooth muscle cells and endothelial cells of arteries with a diameter of approximately 200 *μ*m, which are known to play an important role in pulmonary blood pressure regulation and vascular resistance. Thus, increased VEGF expression in the airway of bronchitis-type patients may lead to increased or even abnormal proliferation of endothelial and vascular smooth muscle cells in pulmonary vessels. Therefore, increased airway VEGF level might closely reflect the magnitude of pulmonary hypertension with exercise in bronchitis-type patients. However, further investigations will be required to confirm our conclusion.

This study has some methodological limitations. First, we had defined the emphysema-type of COPD as apparent LAA by HRCT and the bronchitis-type as little evidence of LAA. However, the HRCT score was significantly higher in bronchitis-type patients than in normal ex-smokers. In this study, bronchitis-type patients had very high cigarette consumption, the reduction in Dlco, and the desaturation after oxygen even breathing supplemental oxygen. A previous study also reported that many patients with bronchitis-type as well as with emphysema-type had very high cigarette consumption [28]. This finding suggests that the sensitivity to smoking in bronchitis-type patients differs from that in emphysema-type patients, in some way. In this regard, hereditary predisposition for dominance of airway disease, but not emphysema, could be an explanation. Moreover, in bronchitis-type patients, obstructive bronchiolitis reduces airflow leading to impairment of gas exchange [29]. In the present study, Dlco was found to decrease in bronchitis-type patients, suggesting that impairment of gas exchange does not occur solely in emphysema-type patients. Moreover, it is plausible to consider bronchitis-type patients as having a mild-degree of emphysematous lesion that might be undetectable as LAA on HRCT scanning. In addition, the relative contribution of parenchymal destruction and small airway disease toward impairment of gas exchange may vary in individuals of bronchitis-type of COPD. Thus, our observation was consistent with those of Gelb et al. who reported 10 cases where pseudophysiological emphysema was caused by severe small airway disease [30]. Therefore, exercise-induced desaturation can occur in patients with bronchitis-type of COPD. Second, our study subjects include moderate to severe COPD patients. Our previous study revealed that VEGF levels in induced sputum were decreased with severity of COPD [31]. In that study, almost study subjects consisted of emphysema-type of COPD, which is a main type of Japanese COPD patients. In the present study, VEGF levels were higher in bronchitis-type patients compared with control subjects. We also found that VEGF level in induced sputum was inversely correlated with FEV1 in bronchitis-type patients, but that its level was not correlated with Dlco and the fall in PaO2 after exercise. Moreover, the significant correlation between the change in mPAP and in PVR during exercise and Dlco could not be observed. These findings suggest that our results are not affected by pathological features of emphysema. Moreover, we could observe the significant correlation between the change in mPAP and in PVR during exercise on room air and the VEGF level. However, exercise challenge on room air may induce hypoxic pulmonary vasoconstriction. Therefore, it is important to perform exercise challenge on oxygen to evaluate the effect of pulmonary vascular remodeling only. In the present study, we attempted to examine the roles of high levels of VEGF in pulmonary vascular remodeling. Therefore, we included patients with bronchitis-type of COPD only. However, to better define the relationship between airway VEGF and pulmonary vascular remodeling, we should also evaluate bronchitis-type patients not developing exercise-induced pulmonary hypertension. Moreover, future studies will be required to determine the direct relationship between morphological analysis of pulmonary vascular remodeling and VEGF level in induced sputum in patients with bronchitis-type of COPD.

## Conclusion

These findings suggest the possibility that VEGF level in induced sputum is a non-invasive marker of pulmonary vascular remodeling in patients with bronchitis-type of COPD. Moreover, our results in the present study may lead to explore mechanisms and treatment of pulmonary vascular remodeling in these patients.

## List of abbreviations used

• ANOVA : one-way analysis of variance

• CI : Cardiac index

• CO : Cardiac output

• COPD : chronic obstructive pulmonary disease

• DLCO : diffusing capacity of the lung for carbon monoxide

• HRCT : high-resolution computed tomographic scans

• LAA : low attenuation area

• PaO2 : arterial oxygen tension

• PAP : pulmonary arterial pressure

• PVR : pulmonary vascular resistance

• PWP : pulmonary wedge pressure

• VEGF : vascular endothelial growth factor

## Competing interests

The author(s) declare that they have no competing interests.

## Authors' contributions

HK participated in the conception and design, acquisition of data, analysis and interpretation of data, and drafting of the manuscript. KA participated in the analysis and interpretation of data, technical support, and critical revision of the manuscript. SN participated in the analysis and interpretation of data, technical support, and critical revision of the manuscript. The article has not been submitted elsewhere and all co-authors have read and approved the final manuscript with its conclusions.
